# Investigating the molecular mechanisms of the “Astragalus-Codonopsis” herb pair in treating diabetes: a network pharmacology and bioinformatics approach with molecular docking validation

**DOI:** 10.3389/fbioe.2025.1618575

**Published:** 2025-07-07

**Authors:** Jinliang Yang, Mingyang Li, Ziyue Zhu, Fengling Han, Yanyan Ma, Jinbo Hou, Qingfeng Zhao, Hui Yuan, XiuMei Li

**Affiliations:** ^1^ Department of Traditional Chinese Medicine, General Hospital of Ningxia Medical University, Yinchuan, China; ^2^ School of Pharmacy, Ningxia Medical University, Yinchuan, China; ^3^ College of Life Science and Technology, China Pharmaceutical University, Nanjing, China; ^4^ Dermatology, Lingwu People’s Hospital, Lingwu, China; ^5^ School of Information Science and Engineering, Henan University of Technology, Zhengzhou, China

**Keywords:** Astragalus-Codonopsis, network pharmacology, molecular dynamics, GSK3 β, insulin resistance

## Abstract

Astragalus membranaceus and Codonopsis pilosula are widely used in traditional chinese medicine for the treatment of diabetes because of their notable hypoglycemic pharmacological effects. Studies have indicatedthat the active compounds in the Astragalus-Codonopsis herb pair may exert their hypoglycemic effects through the modulation of the insulin receptor (IRSP) signaling pathway. In this study, the rhamnolitrin and folic acid were confirmed as the key active components in the Astragalus-Codonopsis herb pair that regulate the IRSP, with their synergistic mechanisms in Type 2 Diabetes Mellitus (T2DM) being further systematically explored by network pharmacology combined with DFT theoretical calculation, molecular docking, molecular dynamics simulation and alanine scanning mutation technology. The results suggest that GSK3β is a critical target through which rhamnolitrin and folic acid exert their anti-diabetic effects. Subsequent molecular docking and molecular dynamics simulations confirmed that both active compounds selected in this study can bind stably with the GSK3β protein. Further alanine scanning mutagenesis experiments validated the importance of key amino acid residues in ligand-receptor interactions. Finally, DFT theoretical calculations provided a detailed elucidation of the binding mechanism between the core components (rhamnolitrin and folic acid) and the target protein GSK3β. This study not only revealed the molecular mechanism of Astragalus-Codonopsis for the treatment of type 2 diabetes, provided a theoretical basis for its clinical application, but also provided a potential molecular target for the development of new anti-diabetes drugs.

## 1 Introduction

The Insulin Receptor Signaling Pathway (IRSP) is a crucial signaling pathway through which cells respond to insulin stimulation, primarily regulating glucose uptake, metabolism and storage (Taha and Klip, 1999; Cantley, 2002). Glycogen Synthase Kinase 3β (GSK3β) is a key protein kinase in the IRSP and plays a significant role in the pathogenesis of Type 2 Diabetes Mellitus (T2DM) (Chen et al., 2024; Sharma et al., 2008). Under normal physiological conditions, GSK3β regulates Glycogen synthesis and breakdown through Glycogen Synthase (GS) (Patel et al., 2008). However, in patients with type 2 diabetes, the activity of GSK3β is abnormally elevated, leading to excessive inhibition of GS activity, reduced glycogen synthesis, and consequently, elevated blood glucose levels, a phenomenon known as insulin resistance (Xi et al., 2016; Zhang et al., 2014). Therefore, inhibition of GSK3β activity to modulate insulin resistance has become one of the important strategies for treating Type 2 Diabetes.

Astragalus membranaceus and Codonopsis pilosula, as traditional herbal medicines, have a long history and wide application in the treatment of diabetes and its complications (Hong et al., 2024; Mao et al., 2022). Modern pharmacological studies have demonstrated that Astragalus-Codonopsis herb pair exhibits significant hypoglycemic effects, potentially through mechanisms such as modulating insulin resistance, improving pancreatic β-cell function, and inhibiting inflammation (Zheng et al., 2020; Lan et al., 2023). Astragalus polysaccharides, as the core active component of Astragalus, have anti-diabetic effects. Relevant studies have shown that the improvement of insulin resistance and islet cell function by astragalus polysaccharides may be the potential mechanisms for the treatment of diabetes (Ke et al., 2017; Yang et al., 2020). Among them, rhamnocitrin and folic acid are considered to be the core active ingredients in Astragalus-Codonopsis pairs, which have potential anti-diabetic effects (El-Khodary et al., 2022). The relevant research evidence suggests that the antioxidant effect of scavenging free radicals from rhamnocitrin may be a potential therapeutic mechanism for diabetes management (Jiang et al., 2008; Jiang et al., 2009; Tu et al., 2007; Zhang et al., 2020). However, the molecular mechanisms by which these components collaboratively exert hypoglycemic effects, particularly their interactions with the GSK3β protein, have not yet been fully elucidated.

In recent years, the emergence of computational biology methods such as network pharmacology, molecular docking and molecular dynamics simulation has provided new approaches for revealing the mechanism of action of multi-components and multi-targets of traditional Chinese medicine (Dupuy et al., 2015). These technologies would enable the analysis of interactions between effective components of traditional Chinese medicines and disease-related targets at a system-wide level, and offer powerful tools for the modernization of traditional Chinese medicine (Li et al., 2023; Pinzi and Rastelli, 2019; Filipe and Loura, 2022). Based on this, the present study aims to systematically explore the inhibitory effects of rhamnocitrin and folic acid on GSK3β in the Astragalus-Codonopsis herb pair and their potential mechanisms for treating Type 2 Diabetes, employing network pharmacology, molecular docking, molecular dynamics simulations, and alanine scanning mutagenesis experiments, with the goal of providing new theoretical insights and experimental support for the modernization of traditional Chinese medicine in diabetes treatment research.

## 2 Materials and methods

### 2.1 Network pharmacological study of Astragalus and Codonopsis in the treatment of T2DM

#### 2.1.1 Enrichment of potential bioactive compounds and targets in Huangqi-Dangshen

Based on the Traditional Chinese Medicine Systems Pharmacology Database and Analysis Platform (TCMSP) (http://lspnwu.edu.cn/tcmspphp), potential bioactive compounds and their corresponding targets in Huangqi and Dangshen were identified according to the criteria of oral bioavailability (OB ≥ 0.30) and drug-likeness index (DL ≥ 0.18) (Ru et al., 2014). The SMILES identifiers of the compounds were retrieved from the PubChem database (https://pubchem.ncbi.nlm.nih.gov/), and further exploration of potential targets not included in the TCMSP platform was conducted using Swiss Target Prediction (http://swisstargetprediction.ch/) (Daina et al., 2019). Subsequently, supplementary screening was performed using the Herb database (http://herb.ac.cn) based on the Lipinski’s Rule of Five, with the following criteria: molecular weight (MW ≤ 500 Da), octanol-water partition coefficient (Alg^p^ ≤ 5), hydrogen bond donors (Hdon ≤ 5), hydrogen bond acceptors (Hacc ≤ 10), and rotatable bonds (RBN ≤10). Next, target screening was carried out using the Batman database (http://bionet.ncpsb.org.cn/batman-tcm/) according to the following criteria: score cutoff (≥0.84), druggability score (≥0.10), and P-value (≤0.05). Finally, the selected target names were converted to standard gene symbols using the UniProt database (https://www.uniprot.org).

#### 2.1.2 Collection and screening of candidate targets for diabetes

The diabetes-related targets were collected using “Diabetes mellitus type 2” and “diabetes” as keywords through GeneCards (https://www.genecards.org/), Therapeutic Target Database (https://idrblab.org/ttd), the Human Mendelian Inheritance Database (https://omim.org/), and the Disgenet database (http://www.disgenet.org/) (Zhou et al., 2024). Therapeutic Target Database (https://idrblab.org/ttd), the Human Mendelian Inheritance Database (https://omim.org/), and the Disgenet database (http://www.disgenet.org/).

#### 2.1.3 Construction of the drug-compound-intersection target regulatory network and identification of core targets

A Venn diagram was constructed using the online tool Draw Venn Diagram (http://bioinformatics.psb.ugent.be) to identify the intersection targets between the drug and diabetes. The identified intersection targets were then imported into the Search Tool for the Retrieval of Interacting Genes/Proteins (STRING) database (Szklarczyk et al., 2023) (https://string-db.org/), with the species set to “*Homo sapiens*” and the protein interaction confidence threshold set to ≥0.40 to filter out nodes lacking interaction. The screening results were exported and a drug-compound-target regulatory network was constructed in Cytoscape 3.8.0. In this network, drugs, compounds, and targets were represented by red diamonds, blue circles, and green triangles, respectively, with the edge weight reflecting the degree centrality of the nodes. Degree centrality analysis of the network was performed using the CytoNCA plugin to calculate the degree centrality values of the core compounds, which were then ranked to identify the core compounds.

#### 2.1.4 Construction of the protein-protein interaction network and identification of core proteins

The drug and diabetes common targets were imported into the STRING platform (https://cn.string-db.org/) to construct the Protein-Protein Interaction (PPI) Network (The network was constructed with “*Homo sapiens*” as the species, confidence set at 0.90, and isolated nodes were removed). The common target network data obtained from the STRING platform was then imported into Cytoscape 3.9.1, where the cytoHubba plugin was used to analyze five network topological parameters of the PPI network: Degree Centrality (DC), Betweenness Centrality (BC), Closeness Centrality (CC), Maximal Clique Centrality (MCC), and Maximum Neighborhood Component (MNC). The average value for each parameter was calculated, and data exceeding the average values were selected for Venn analysis, followed by the construction of the corresponding PPI network diagram. Subsequently, targets exceeding the average values of each parameter were then displayed and identified as the core targets of the “Astragalus-Dangshen” herbal pair in the treatment of T2DM.

#### 2.1.5 Cluster analysis

In order to identify the core sub-clusters within the PPI network, this study employed the MCODE plugin in Cytoscape 3.9.1 to perform cluster analysis on the intersected target network. The filter conditions were set as follows: Degree Cutoff = 2, K-core = 2, and Max. Depth = 100.

#### 2.1.6 Gene ontology (GO) and kyoto encyclopedia of genes and genomes (KEGG) pathway enrichment analysis

Finally, GO and KEGG pathway (Kanehisa et al., 2017; Kanehisa et al., 2023) enrichment analysis were performed by ShinyGO 0.82. All enrichment analyses were conducted in the “*Homo sapiens*” species, with results selected at P < 0.01 to identify biological processes (BP), cellular components (CC), molecular functions (MF), and KEGG signaling pathways associated with glucose regulation, thereby further exploring the potential mechanisms of “Huangqi-Dangshen” medicinal pair in blood glucose regulation.

### 2.2 Molecular docking

The diabetic-related receptor proteins identified in Section 2.1 were preprocessed using Discovery Studio 2019 win, which primarily involved repairing missing residues, optimizing protonation states, and removing crystallization water molecules to ensure the structural integrity of the receptor. Then, using the semi-flexible docking method in the CDOCKER module (Tessaro and Scapozza, 2020), the residues within a 10 Å range of the eutectic ligand were defined as the active pocket, and the binding process between the ligand and receptor protein was simulated. In the end, according to the energy score and screening results, the best binding mode was selected as the initial conformation of the subsequent molecular dynamics simulation.

### 2.3 Molecular dynamics simulation

Based on the results from Section 2.2, this study performed molecular dynamics simulations to investigate the binding of rhamnocitrin and folic acid with the target protein GSK3β. Molecular dynamics simulation can simulate the movement and interaction of molecules at the atomic level, and analyze the dynamic changes of proteins, ligands and the environment, so as to provide information on the behavior of molecular conformational change, binding stability and dynamics between protein and ligand. In this study, Schrodinger 2024 Linux program was used to construct a molecular dynamics simulation system. In the simulation system, the receptor-ligand complex was placed in a TIP3P water solvent model, with the box boundary set at least 12 Å away from the complex to ensure adequate solvation of water molecules and avoid boundary effects. The ion concentration in the system was set to 0.154 M, with Na^+^ and Cl^−^ used to neutralize the charge of the system in the simulation process. To enhance simulation accuracy, the OPLS3e force field (Tirado-Rives and Jorgensen, 2008) was employed in the system, which effectively describes non-bonded interactions and binding modes between molecules, particularly suited for the study of protein-ligand complexes (Wu et al., 2022; Collier et al., 2020).

In the initial energy optimization, the steepest descent method was adopted for 5,000 steps to minimize unreasonable contact and high-energy conformations, with a convergence threshold set at 10 kJ/mol/nm to ensure effective adjustment of the force field parameters. Then the conjugate gradient method was applied for 2000 steps to optimize the thermodynamic state of the system in an effort to guarantee its stability (Levitt and Lifson, 1969; Powell, 1977; Fletcher, 2013).

When entering the equilibrium phase, the NVT ensemble simulation of 100 ps with a time step of 2 fs was first performed, and the system was gradually heated to 300 K so as to eliminate the influence of the initial structure and make sure thermodynamic equilibrium. This was followed by switching to the NPT ensemble for further equilibration at a constant pressure of 1 bar, with a simulation time of 100 ps to stabilize the density and pressure. The primary purpose of this stage was to allow the system to reach a stable thermodynamic state, preparing it for subsequent production simulations (Zhao et al., 2007; Wen et al., 2003).

The formal molecular dynamics simulations ran for 100 ns, during which the temperature was maintained at 300 K, the pressure at 1 bar, with the time step of 2 fs and trajectory sampling interval of 10 ps. In order to ensure the stability and accuracy of the simulation, physical properties such as temperature, pressure and volume were regularly monitored during simulation process to ensure they remained within the expected range.

### 2.4 Alanine flexible scanning

On the basis of molecular dynamics simulation, in order to further analyze the interactions between ligands and key residues of diabetes-related target proteins, the Discovery Studio 2021 program was utilized in this study to carry out alanine flexible scanning. Alanine scanning replaces critical residues with alanine to identify residues that play a crucial role in ligand binding, and to assess their impact on the binding interface and affinity. The scanning targets are all amino acid residues within a 3 Å radius of the ligand-binding interface.

### 2.5 Theoretical calculation of DFT

In this study, density functional theory (DFT) (Zhao et al., 2004; Qin et al., 2018) was used to evaluate the chemical activity of Astragalus-Codonopsis drugs on core components. Firstly, the geometric structures were optimized at the B3LYP/6-311G D3 (d, p) level (Harder et al., 2016)by the using of Gaussian 09w Linux program. After optimization, single-point energy calculations were performed under the same basis set, and energy distribution data for the Highest Occupied Molecular Orbital (HOMO) and the Lowest Unoccupied Molecular Orbital (LUMO) were extracted (Zhang and Musgrave, 2007). By analyzing the HOMO-LUMO gap and orbital spatial distribution, the electronic excitation properties of the core components and their electron transfer activity at the GSK3β protein binding interface were evaluated. In addition, the HOMO-LUMO orbital distribution and electron density plots were analyzed and visualized using Multiwfn_3.8 (Lu and Chen, 2012; Lu, 2024).

## 3 Results

### 3.1 Analysis of the results of network pharmacology study of Astragalus-Codonopsis in the treatment of diabetes

#### 3.1.1 Intersection gene targets analysis

**FIGURE 1:**
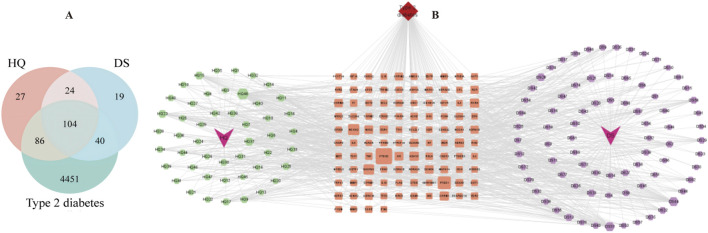
**(A)** Common targets for diseases and drugs. **(B)** Traditional Chinese Medicine-active ingredients-target genes.

Based on the research methodology outlined in Section 2.1, a total of 70 potential bioactive compounds and 300 associated drug targets from the Astragalus- Codonopsis pharmaceutical pair were identified. Subsequently, 4,681 disease-related targets associated with Type 2 Diabetes were obtained from disease databases, and through cross-analysis, 104 drug-disease target intersections were selected (Figure 1A). Additionally, a “Traditional Chinese Medicine-Active Compounds-Target Network” was successfully constructed by Cytoscape 3.9.1 (Shannon et al., 2003) (Figure 1B). Based on this, 95 nodes and 618 interaction relationships were obtained. Moreover, 12 key active compounds were further selected based on their degree values (Table 1) by CytoNCA plug-in of Cytoscape 3.9.1.

**TABLE 1:** Degree values of core drugs ingredients.

Compound	Degree	CAS	Compound	Degree	CAS
Rhamnocitrin	79	569–92-6	Mucronulatol	25	20,878–98-2
Folic acid	56	59–30-3	Tangshenoside III_qt	22	129,277–39-0
Isorhamnetin	56	480–19-3	7-O-Methylisomucronulatol	21	137,217–83-5
Glycitein	37	40,957–83-3	Tectorigenin	21	548–77-6
Calycosin	28	20,575–57-9	7-Methoxy-2-methyl isoflavone	18	19,725–44-1
Formononetin	25	485–72-3	1-(2,4-Dihydroxyphenyl)-3-	19	961–29-5
			(4-Hydroxyphenyl) prop-2-en-1-one		

#### 3.1.2 PPI network analysis of key targets of anti-T2DM

**FIGURE 2:**
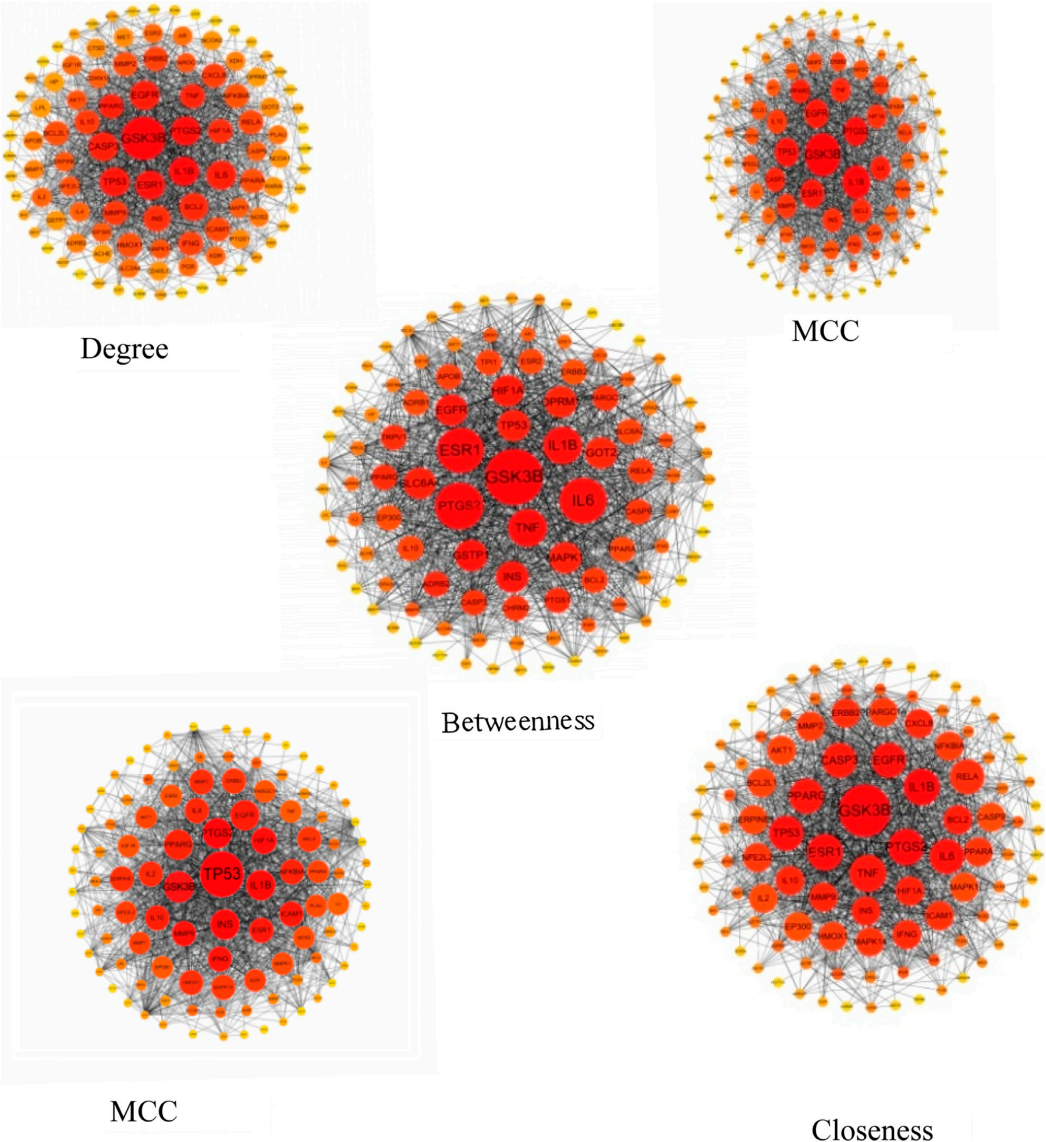
The PPI network for 104 overlapping genes (The size and color of the nodes were positively correlated with the target’s degree of association.).

**FIGURE 3:**
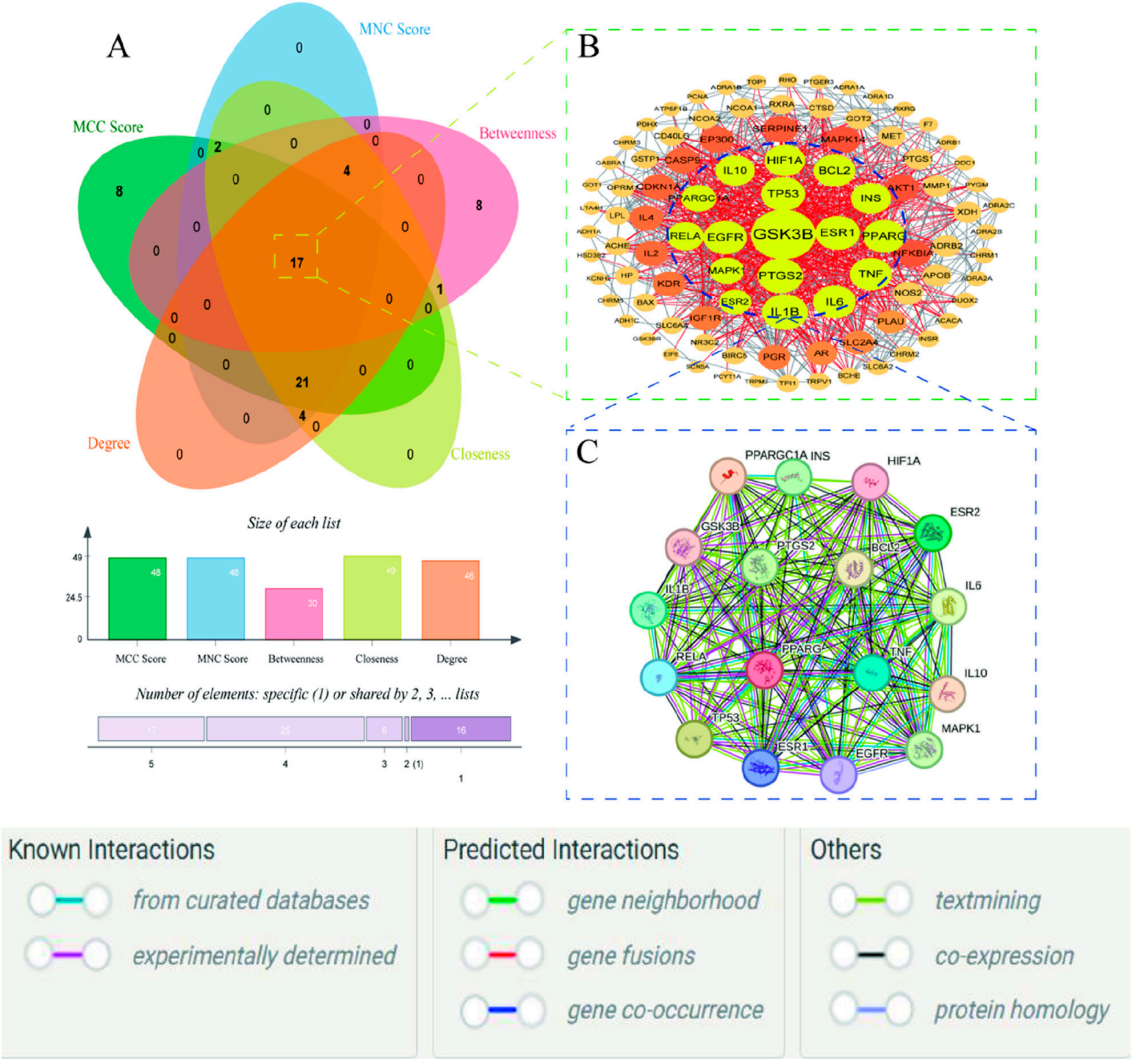
**(A)** The venn diagram for MCC, MNC, DC, CC and BC **(B)** 104 common targets PPI network. The highlighted targets originate from the key intersecting nodes determined by the preceding Venn diagram analysis **(C)** PPI network of the key genes; PPI network by the screening criteria of DC ≥ 26.29, BC ≥ 100.85, CC ≥ 0.534090909, MCC ≥ 88570.58, MNC ≥ 25.32.

The five network topological parameters of the intersected target network were calculated using the “cytoHubba” plugin. PPI network diagrams associated with these five parameters were subsequently plotted (Figure 2). Through Venn analysis, 17 core targets were identified, including GSK3B, TP53, PTGS2, INS, IL1B, among others (Figure 3A; Table. 2). Finally, the core protein-protein interaction network of the “Astragalus-Dangshen” herbal pair in the treatment of T2DM was visualized (Figures 3B,C).

**TABLE 2:** Analysis of the network topological parameters of key targets.

NO.	Gene name	Betweenness	Closeness	MNC score	MCC score	Degree
1	GSK3B	1,075.30	0.74	71	1.05 × 10^16^	104
2	PTGS2	701.91	0.71	62	1.05 × 10^16^	63
3	ESR1	574.35	0.69	59	1.04 × 10^16^	61
4	TNF	464.45	0.66	55	5.33 × 10^12^	56
5	IL6	363.12	0.68	57	2.65 × 10^11^	57
6	IL1B	349.15	0.71	63	1.05 × 10^16^	64
7	TP53	282.42	0.67	60	1.05 × 10^16^	60
8	INS	291.48	0.65	52	1.05 × 10^16^	52
9	EGFR	291.25	0.67	58	1.04 × 10^16^	59
10	HIF1A	286.13	0.63	52	1.05 × 10^16^	53
11	PPARG	217.35	0.66	57	8.37 × 10^15^	57
12	MAPK1	249.61	0.58	39	8.92 × 10^13^	39
13	RELA	148.25	0.61	45	4.43 × 10^15^	45
14	ESR2	114.67	0.57	34	5.69 × 10^12^	35
15	BCL2	148.47	0.65	54	5.31 × 10^8^	55
16	IL10	132.13	0.63	49	1.04 × 10^16^	50
17	PPARGC1A	192.80	0.59	41	8.37 × 10^15^	41

#### 3.1.3 Cluster analysis

**FIGURE 4:**
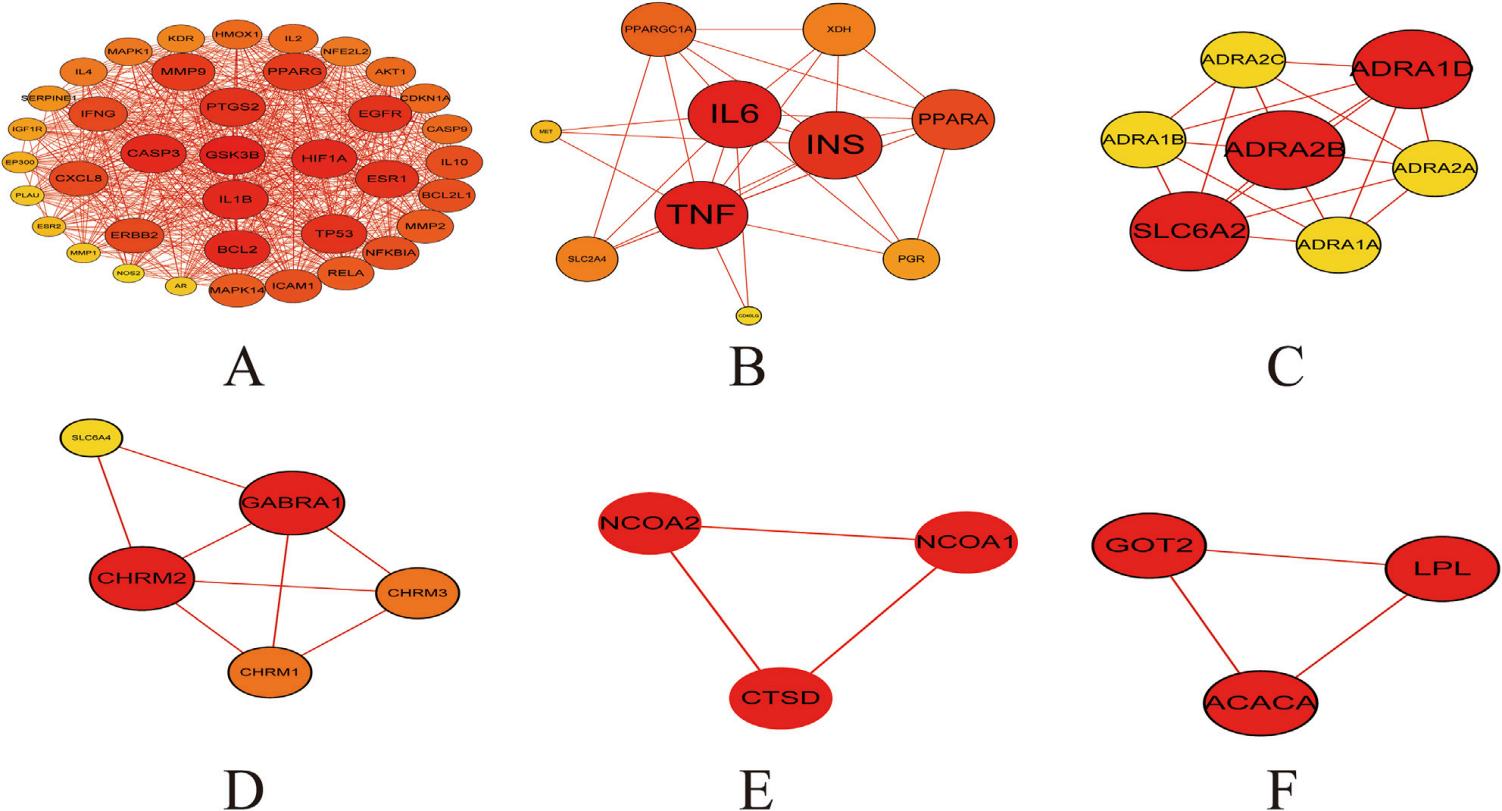
Cluster analysis of the overlapping protein-protein interaction (PPI) network comprising 104 nodes **(A)** Cluster 1 contains 38 nodes connected by 620 edges (cluster score = 32.21) **(B)** Cluster 2 consists of 10 nodes with 29 edges (score = 6.44) **(C)** Cluster 3 includes 7 nodes and 19 edges (score = 6.33) **(D)** Cluster 4 comprises 5 nodes and 8 edges (score = 4.00) **(E,F)** Cluster 5 and 6 each contain 3 nodes connected by 3 edges (score = 3.00) The size and color of the nodes were positively correlated with the target’s degree of association.

Cluster analysis revealed that the PPI network could be partitioned into six distinct modules (Figures 4A–F; Table 3). Module 1 emerged as the central hub of the PPI network, exhibiting a significantly higher cluster score (score = 34.15) compared to other modules. Notably, all core targets identified in Method 1.1.2 were localized within Module 1, further underscoring the pivotal role of these targets in the therapeutic mechanism of the Astragalus-Codonopsis herb pair for T2DM management.

**TABLE 3:** Cluster information of the common targets for diseases and drugs.

Cluster	Score	Nodes	Edges	Node IDs
1	32.21	38	620	IL10, EP300, PPARG, MMP1, IL4, NFE2L2, TP53, IGF1R, BCL2L1, EGFR, AR, HIF1A, CXCL8, NFKBIA, IL1B, PLAU, ESR1, IFNG, MAPK14, HMOX1, MMP9, CASP3, IL2, SERPINE1, GSK3B, AKT1, CDKN1A, ESR2, MMP2, ERBB2, PTGS2, CASP9, RELA, NOS2, MAPK1, ICAM1, BCL2, KDR
2	6.44	10	29	SLC2A4, TNF, XDH, PPARA, IL6, INS, PGR, MET, CD40LG, PPARGC1A
3	6.33	7	19	ADRA2C, SLC6A2, ADRA2B, ADRA1A, ADRA2A, ADRA1D, ADRA1B
4	4.00	5	8	GABRA1, SLC6A4, CHRM1, CHRM3, CHRM2
5	3.00	3	3	CTSD, NCOA2, NCOA1
6	3.00	3	3	GOT2, ACACA, LPL

#### 3.1.4 Functional enrichment analysis of intersection target of Astragalus-Codonopsis pairs and T2DM

**FIGURE 5:**
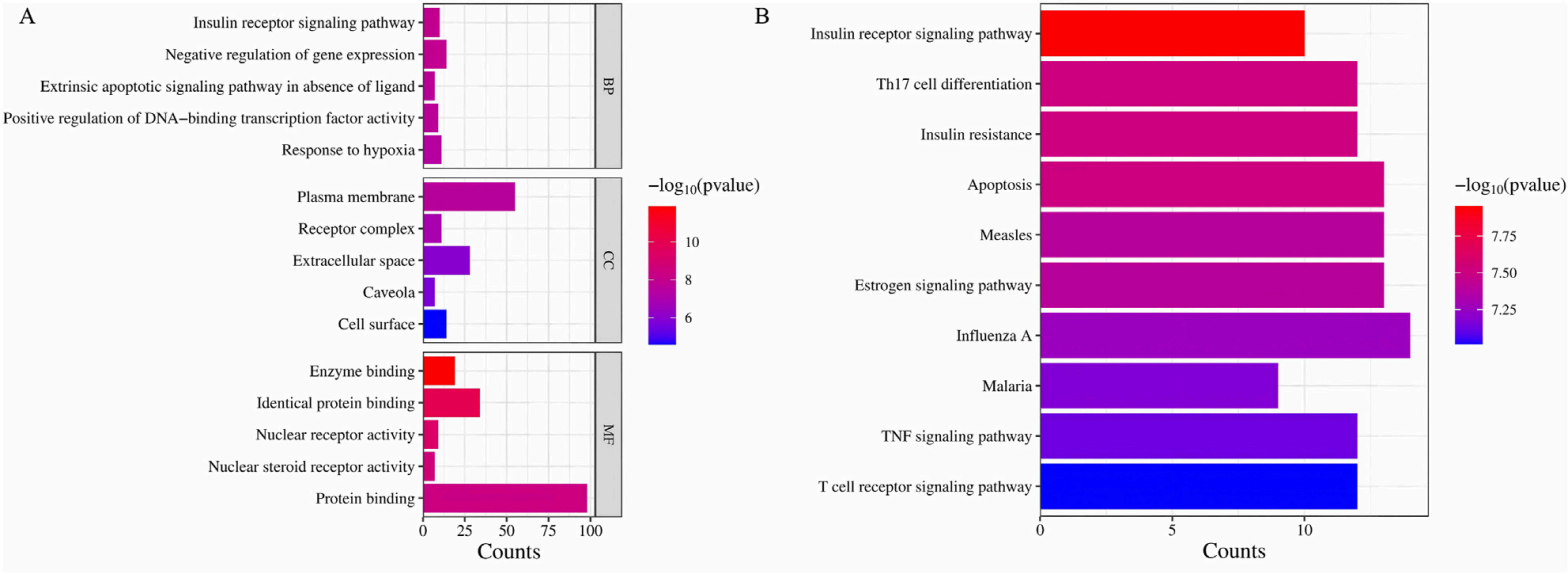
**(A)** GO enrichment analysis **(B)** KEGG pathway enrichment analysis ingredients-target genes.

The functional enrichment analysis was performed on the 104 potential target genes identified by the use of DAVID 6.8 database (Sherman et al., 2022; Huang et al., 2009) which included GO enrichment analysis and KEGG pathway analysis. The GO analysis primarily includes three components: stands for Biological Process (BP), stands for Cellular Component (CC), and stands for Molecular Function (MF). The top 15 pathways with the smallest p-values from the gene enrichment analysis (Figure 5A) and the top 30 KEGG pathways with the smallest p-values (Figure 5B) were visualized. The results indicated that the IRSP is a key pathway through which the Astragalus-Codonopsis pharmaceutical pair regulates Type 2 Diabetes Mellitus (T2DM). Based on the above research content, we propose that the main bioactive compounds of the Astragalus- Codonopsis pharmaceutical pair regulate blood glucose levels by binding to GSK3β protein and modulating the expression of the insulin receptor signaling pathway.

### 3.2 Results of molecular docking between Astragalus-Codonopsis pair and core target GSK3β

**FIGURE 6:**
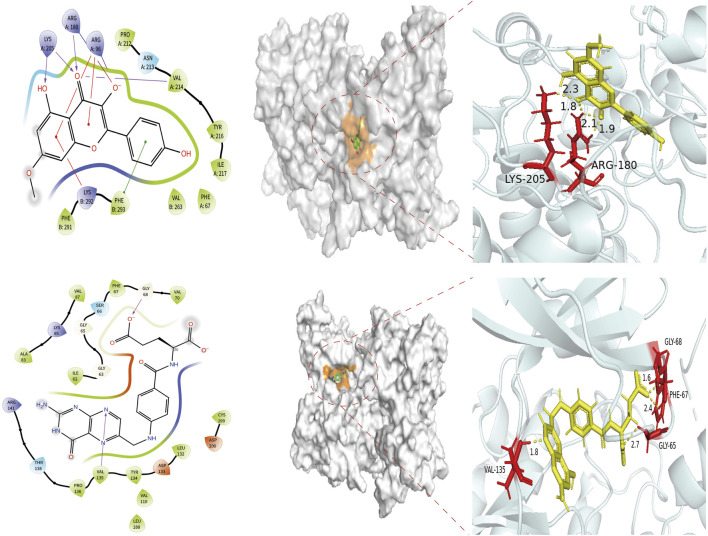
Molecular docking of target protein GSK3β and two screened key ingredients. Docking diagram of rhamnocitrin and folic with protein GSK3β.

In this study, the binding pattern and affinity of 13 core components from Astragalus and Codonopsis Radix with their core targets were evaluated by molecular docking technology. Ultimately, two ligand-protein complexes with stronger binding effects were selected and assessed. As shown in Figure 6, among the 13 core compounds, compound Rh (rhamnocitrin) exhibited relatively stable binding with the GSK3β protein, especially the formation of hydrogen bonds with residues Lys205 and Asn213, and interacting with other amino acid residues through van der Waals forces, resulting in a docking score of 119.1087. This indicates that it has strong binding affinity. In contrast, the binding of compound Fa (folic acid) with protein GSK3β involved more amino acid residues, forming multiple hydrogen bonds between Ser66, Gly63, Phe67, Gly65, and Asp133, with a docking score of 130.2159, indicating higher binding stability.

### 3.3 Alanine flexible scanning

The alanine scanning results indicate that the binding energies of mutations at residues Arg180, Lys165 and Val214 on chain A, and Lys292 and Phe293 on chain B, were all greater than 0.5 after alanine substitution for the Rh-GSK3β complex (Table 4). This suggests that the binding interaction between GSK3β protein and rhamnocitrin was weakened, leading to a disruption in the stability of the complex after the mutation. Similarly, in the Fa-GSK3β complex (Table 5), when the residues Gly65, Val70, Lys85, and Leu132 were all replaced with alanine on protein chain A, the binding energies increased to varying degrees, which meant that the stability of the complex was more destroyed. These results suggest that the aforementioned amino acids play a magnificent role in the binding process between folate and the key protein GSK3β, which is consistent with the previous molecular docking findings.

**TABLE 4:** Virtual alanine scanning mutagenesis of the rhamnocitrin-protein GSK3β.

GSK3β-Rh-mutation	Mutation energy^kcal/mol^	Effect of mutation	VDW term	Electrostatic term	Entropy
A: Phe67 > ALA	0.31	Neutral	0.73	−0.07	−0.02
A: Arg180 > ALA	1.57	Destabilizing	2.00	0.79	0.22
A: Lys205 > ALA	1.60	Destabilizing	2.04	0.69	0.29
A: Pro212 > ALA	0.10	Neutral	0.16	0.04	0.00
A: Asn213 > ALA	0.19	Neutral	0.47	−0.11	0.01
A: Val214 > ALA	0.55	Destabilizing	1.28	−0.11	−0.04
A: Ile217 > ALA	0.37	Neutral	0.66	0.04	0.02
B: Val263 > ALA	0.37	Neutral	0.76	−0.05	0.02
B: Lys292 > ALA	0.58	Destabilizing	2.67	−0.62	−0.55
B: Phe293 > ALA	1.18	Destabilizing	2.40	−0.1	0.04

**TABLE 5:** Virtual alanine scanning mutagenesis of the folic acid-protein GSK3β.

GSK3β-Fa mutation	Mutation energy^kcal/mol^	Effect of mutation	VDW term	Electrostatic term	Entropy
A: Gly65 > ALA	3.05	Destabilizing	5.34	0.76	0.00
A: Ser66 > ALA	−0.17	Neutral	−0.24	−0.12	0.01
A: Phe67 > ALA	0.18	Neutral	0.62	−0.18	−0.05
A: Gly68 > ALA	−0.27	Neutral	−0.57	0.07	−0.02
A: Val70 > ALA	0.68	Destabilizing	1.54	−0.24	0.04
A: Ala83 > ALA	0.05	Neutral	0.12	−0.02	0.00
A: Lys85 > ALA	1.69	Destabilizing	2.03	1.11	0.15
A: Val110 > ALA	0.22	Neutral	0.42	0.02	0.00
A: Leu132 > ALA	0.63	Destabilizing	1.18	−0.04	0.08
A:Asp133 > ALA	−0.24	Neutral	0.05	−0.52	0.00
A: Tyr134 > ALA	0.41	Neutral	0.64	0.00	0.11
A: Val135 > ALA	0.03	Neutral	0.11	−0.07	0.01
A: Leu188 > ALA	0.15	Neutral	0.33	−0.05	0.01
A: Cys199 > ALA	0.02	Neutral	0.04	−0.03	0.02
A: Asp200 > ALA	−0.20	Neutral	2.08	−2.41	−0.04

### 3.4 Molecular dynamics simulation results

**FIGURE 7:**
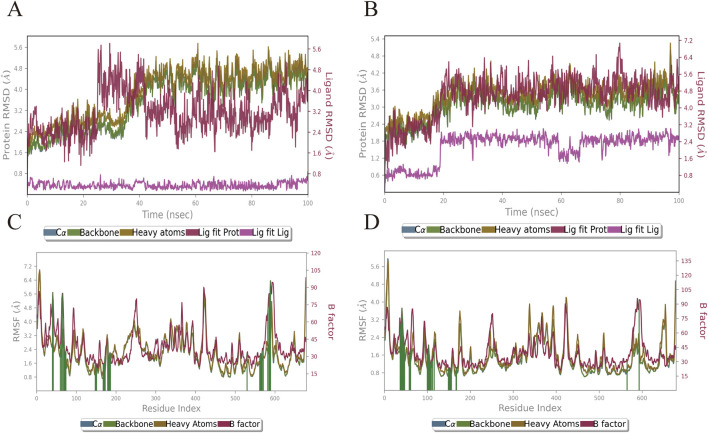
The variation profiles of RMSD and RMSF for the Rhamnocitrin- GSK3β complex and Folic Acid- GSK3β complex. RMSD of the Rhamnocitrin-Protein GSK3β **(A)** and Folic Acid-Protein GSK3β **(B)**; RMSF of the Rhamnocitrin-Protein GSK3β **(C)** and Folic Acid-Protein GSK3β **(D)**.

Based on the results of molecular docking, we simulated the molecular dynamics of two groups of receptor-ligand complexes (Rh-GSK3β, Fa-GSK3β) over a period of 100 ns The complexes formed by rhamnocitrin (Rh) and folic acid (Fa) with GSK3β exhibited structural stability, as demonstrated by molecular dynamics simulations. Figures 7A,B show the changes of RMSD (Root Mean Square Deviation) of the complex formed by the combination of active ingredient Rh and Fa with protein GSK3β in a vivo environment, respectively. In the FA-GSK3β complex, the binding of compound Fa with protein GSK3β shows a significant conformational reversal at the 20th ns, after which the RMSD fluctuation maintained around 3.6 Å steadily, exhibiting good uniformity, and the conformational changes of receptor and ligand were synchronized. The RMSD fluctuation of complex Rh-GSK3β began to rise around the 30th ns, and the fluctuation became stable at the 40th ns. Finally, the conformation changes of protein stabilized around 4 Å, while the conformation change of ligand stabilized around 3.2 Å. Subsequently, we analyzed the changes in RMSF (Root Mean Square Fluctuation) of the two groups of complex systems (Figures 7C,D), the value of which represents the flexibility of the corresponding amino acid residues. The results show that amino acid residues in contact with the core components in contact with the core components exhibit minimal fluctuation, suggesting lower flexibility and higher rigidity, that is, the fluctuation of the residues of the receptor remain relatively stable, and the binding of the core components Rh and Fa to the target protein GSK3β maintain stable, reducing the possibility of deviation due to large fluctuations.

**FIGURE 8:**
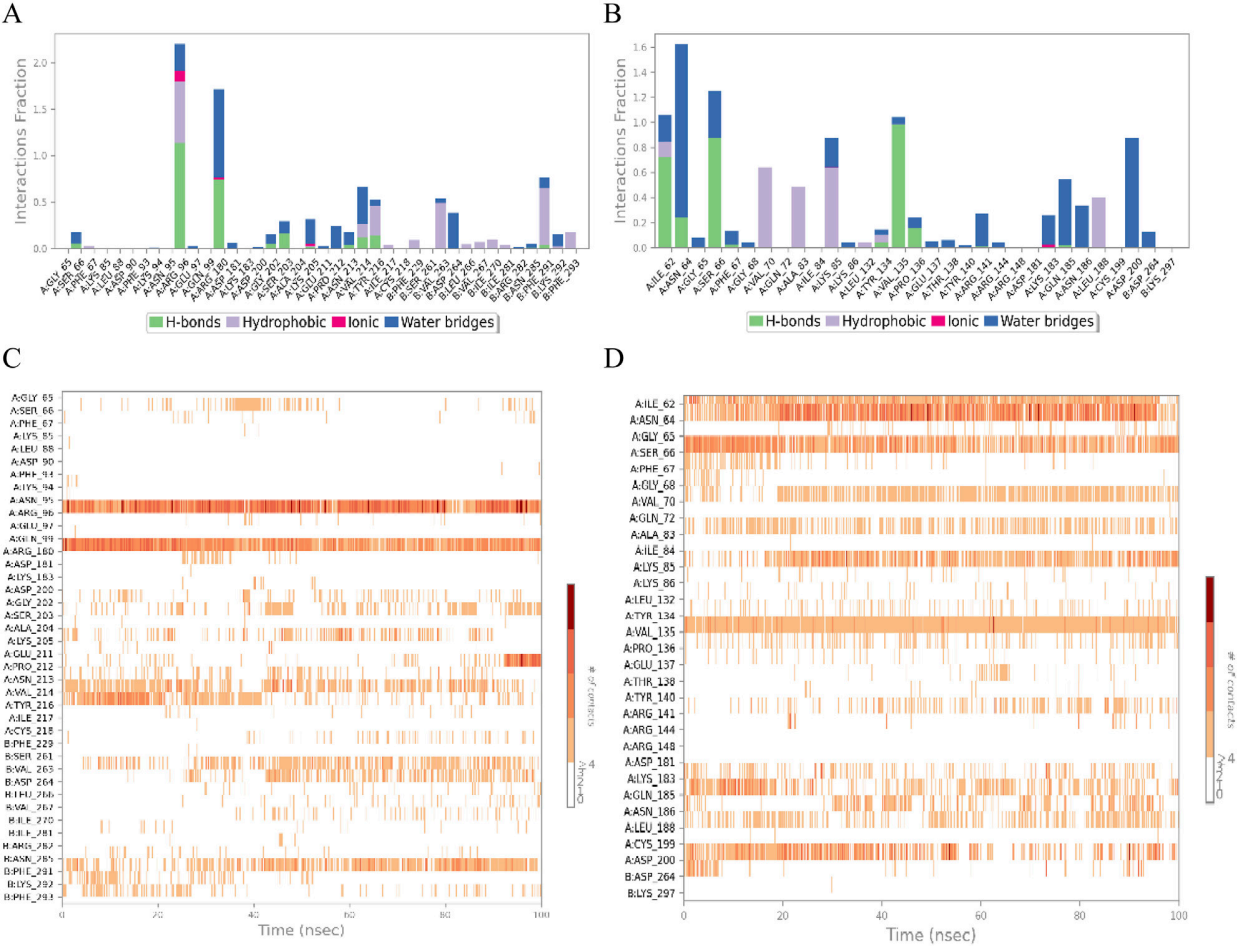
The ligand-receptor binding interface interaction analysis diagram of Rhamnocitrin-Protein GSK3β **(A)** and Folic Acid-Protein GSK3β **(B)**; the hydrogen bond occupancy timeline plot of Rhamnocitrin-Protein GSK3β **(C)** and Folic Acid-Protein GSK3β **(D)**.

In order to further investigate the physical and chemical pathways of selected ligand compounds binding to the receptor active pockets bind, the interaction analysis of the ligand-receptor binding interface was conducted (Figures 8A,B). In the Rh-GSK3β system, the hydrogen bond interaction between the compound Rh and residues of Asn95 and Gln99 is formed, with the duration of this interaction exceeding 100% and 50%, respectively. In addition, the ligand Rh exhibits hydrophobic interactions with residues Val214, Tyr216, Ser261, Val263 and Asn285. In FA-GSK3β system, ligand Fa forms continuous hydrogen bonds with Ile62, Ser66 and Val135 residues, as well as with Asn64, Gln185 and Asp200 residues through water bridges. The hydrogen bond occupancy timeline diagram of the complex also confirmed this point (Figures 8C,D).

**FIGURE 9:**
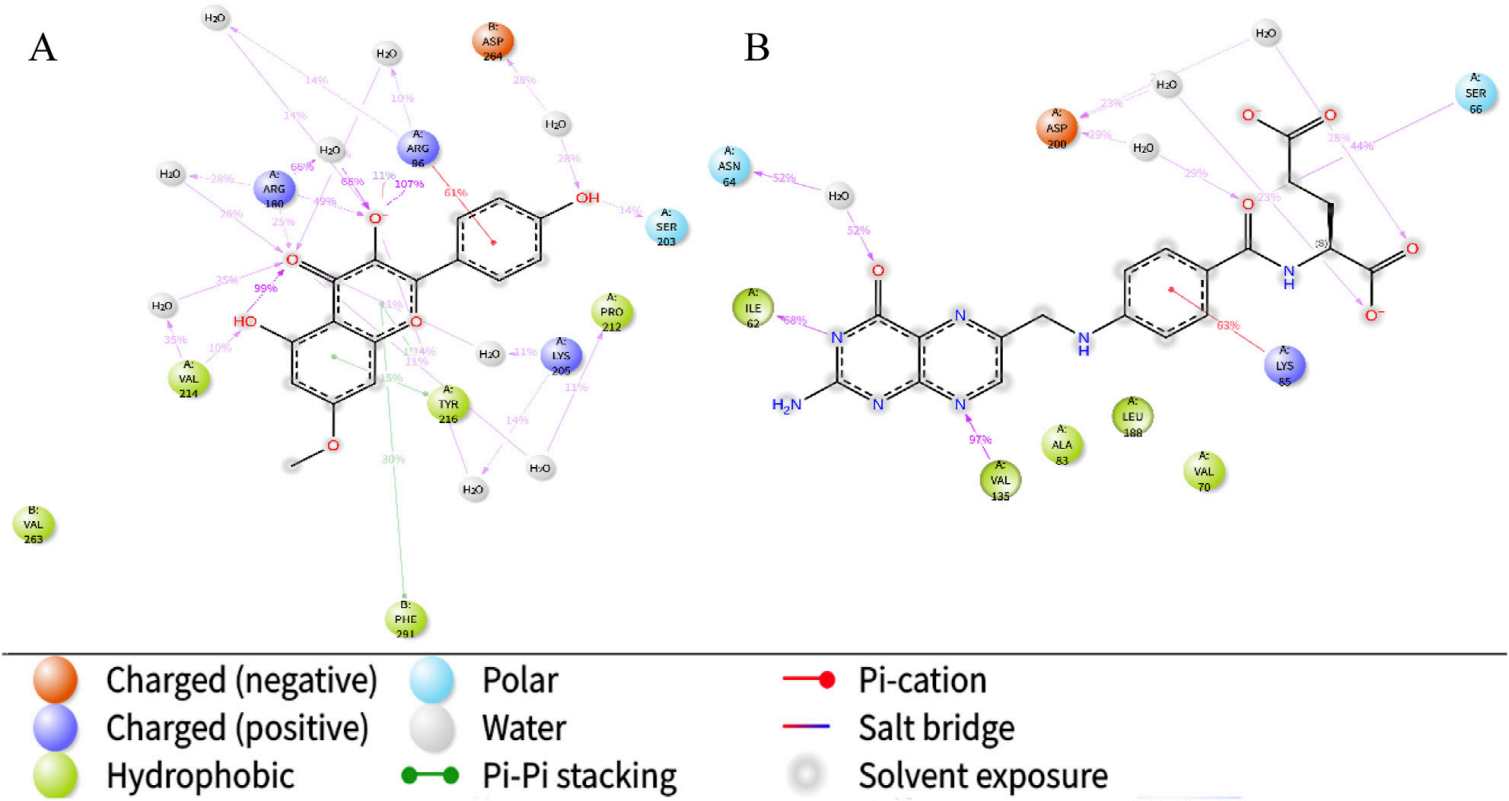
The schematic of detailed ligand atom interactions with protein residues. Interaction diagram of Rhamnocitrin- GSK3β **(A)** and Folic Acid- GSK3β **(B)**.

Next, we specifically focused on investigating the impact of the ligands on the interactions within the complex systems (Figure 9). The results indicate that the carbonyl, hydroxyl, and oxygen ions of the Rh ligand contribute most of the interaction of the complex interface. For example, the carbonyl group interacts with Arg180 residue of the A chain by dispersion-induced interaction in 25% of the total simulation time, while the hydroxyl group forms a water-bridge interaction with the Asp264 residue of chain B, with the interaction duration accounting for 28%. In the FA-GSK3β system, the N and O atoms of the ligand Fa play a crucial role in the binding process of the interface, forming persistent chemical interactions with several amino acid residues (such as water-bridge and salt-bridge interactions).

**FIGURE 10:**
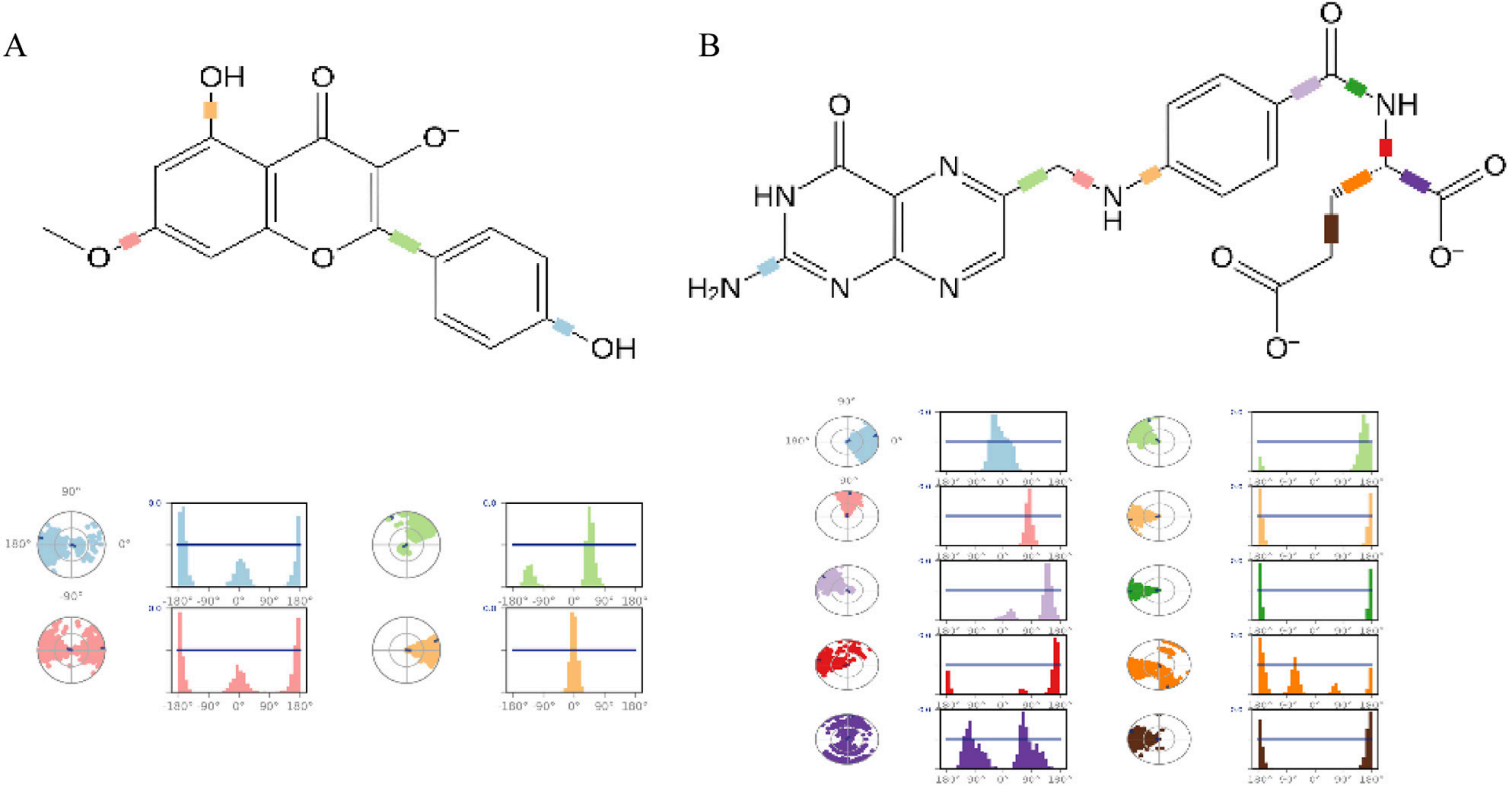
The ligand torsion profile of ligand-protein. The diagram of Rhamnocitrin- GSK3β **(A)** and Folic Acid- GSK3β **(B)**.

Finally, the torsion angle distribution histogram was used to quantitatively analyze the conformational changes of the ligands (Figure 10). The study revealed that the C2-C1-O1-C3 dihedral angle of the Rh ligand exhibits a bimodal distribution (peaks at ± 45°), indicating that the ligand is embedded in small cavities within the receptor pocket, demonstrating significant stability. In the Fa-GSK3β system, the rotation of the C-C bonds at positions 9 and 12 of the Fa ligand is significantly inhibited, suggesting that these portions are inserted into the binding pocket of the GSK3β protein, preventing bond rotation and indicating a stable binding interaction with the protein.

### 3.5 The DFT theoretical calculations of Rh and Fa

**FIGURE 11:**
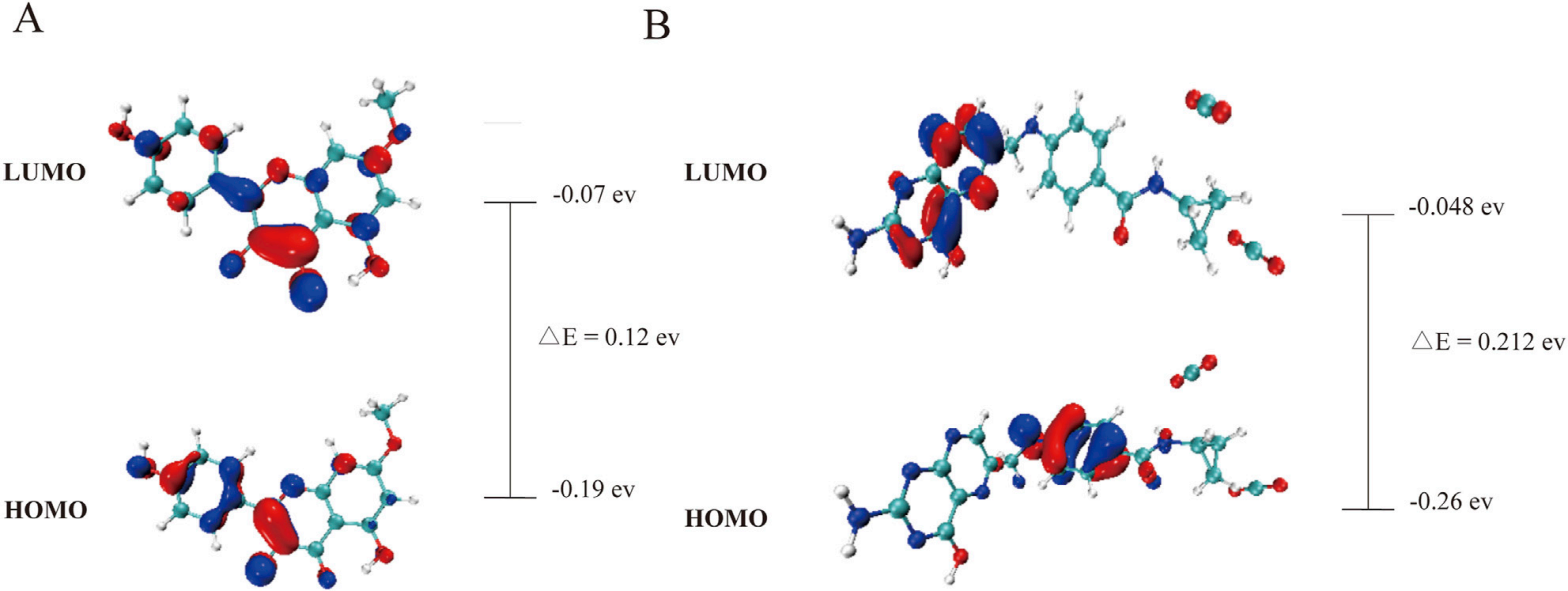
The HOMO-LUMO energy levels of rhamnocitrin **(A)** and folic acid **(B)**.

The computational results (Figure 11) show that the low HOMO-LUMO gaps (0.12 eV for Rh, 0.21 eV for Fa) suggest enhanced electron transfer activity, facilitating interactions with polar residues of GSK3β. The HOMO orbital of rhamnolitrin is predominantly localized on the double bond and hydroxyl group of the pyran ring, as well as the phenolic hydroxyl group of the attached benzene ring, while the LUMO orbital is distributed over the carbon-oxygen double bond of the pyran ring and the carbon-carbon single bond connecting to the benzene ring. This configuration suggests that the electron tends to transfer from the benzene ring to the pyran ring. Furthermore, its low energy gap facilitates electron transfer within the GSK3β protein’s binding pocket, leading to the rearrangement of electron clouds in the polar amino acid residues, thereby strengthening the hydrogen bonding and enhancing the binding stability between rhamnetin and GSK3β. In addition, the delocalized π-electron system in the structure of rhamnolitrin can engage in π-π stacking or cation-π interactions with aromatic residues (such as Phe and Tyr), synergistically enhancing the binding stability. The HOMO orbital of the folic acid molecule is primarily distributed on the amine group and the phenyl ring of the para-aminobenzoic acid, while the LUMO orbital is concentrated on the adjacent pteridine structure. What’s more, the low energy gap also enables electronic transfer potential. It is similar to the results observed for rhamnetin. Both molecules enhance their binding stability to the GSK3β protein through electronic transfer and π-π stacking effects.

**FIGURE 12:**
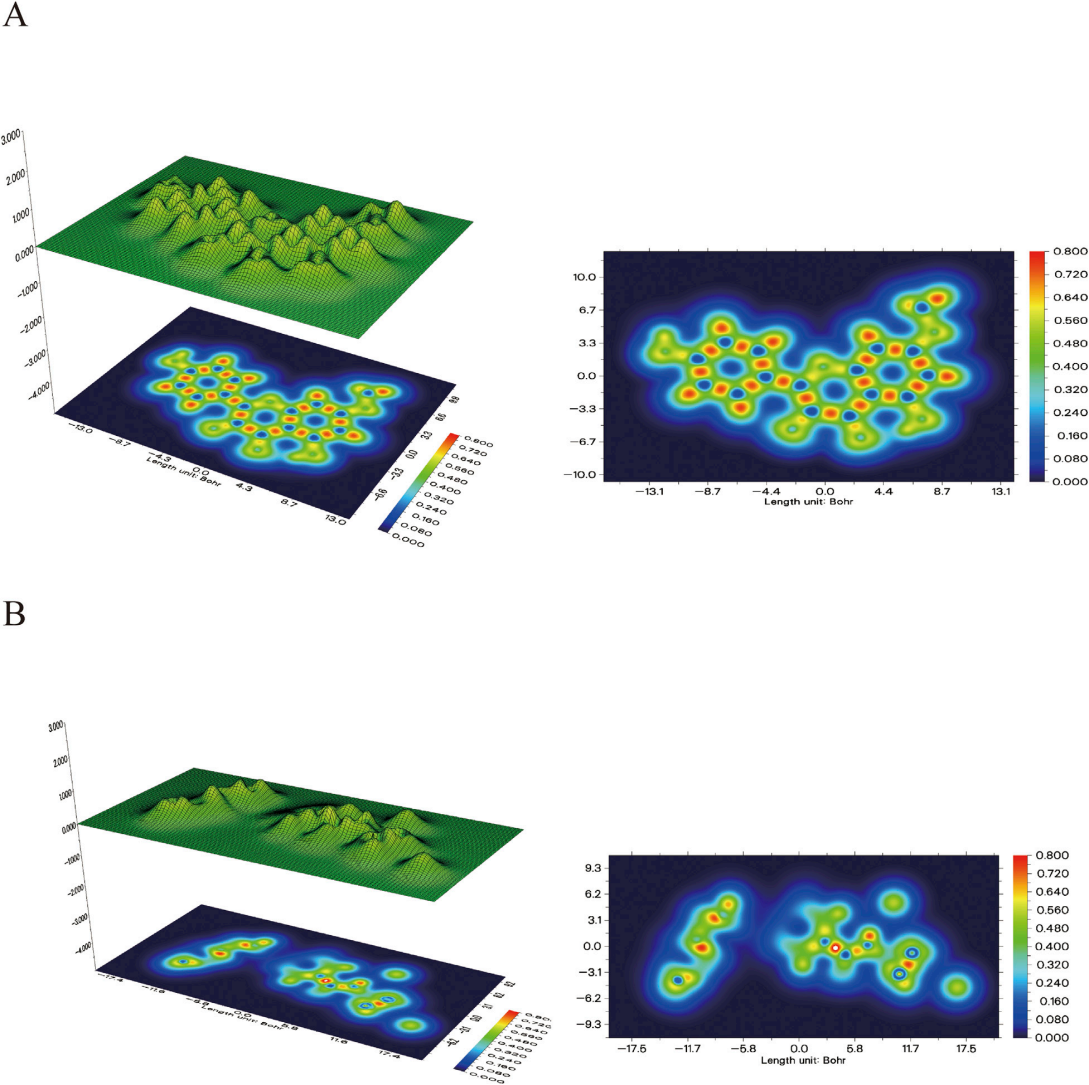
The electron density map and its 3D projection of rhamnocitrin **(A)** and folic acid **(B)**.

In addition, the electron density map in Figure 12 further validates the binging mechanism of rhamnolitrin with target protein GSK3β. The electron density of folic acid is primarily concentrated on the pteridine structure and the carbonyl group of the amide bond, which may serve as potential binding sites for stable interaction with the target protein. The electron density distribution of rhamnolitrin is uniform, suggesting that its binding to GSK3β primarily relies on the delocalized π-electrons and the π-π accumulation with amino acid residues.

**FIGURE 13:**
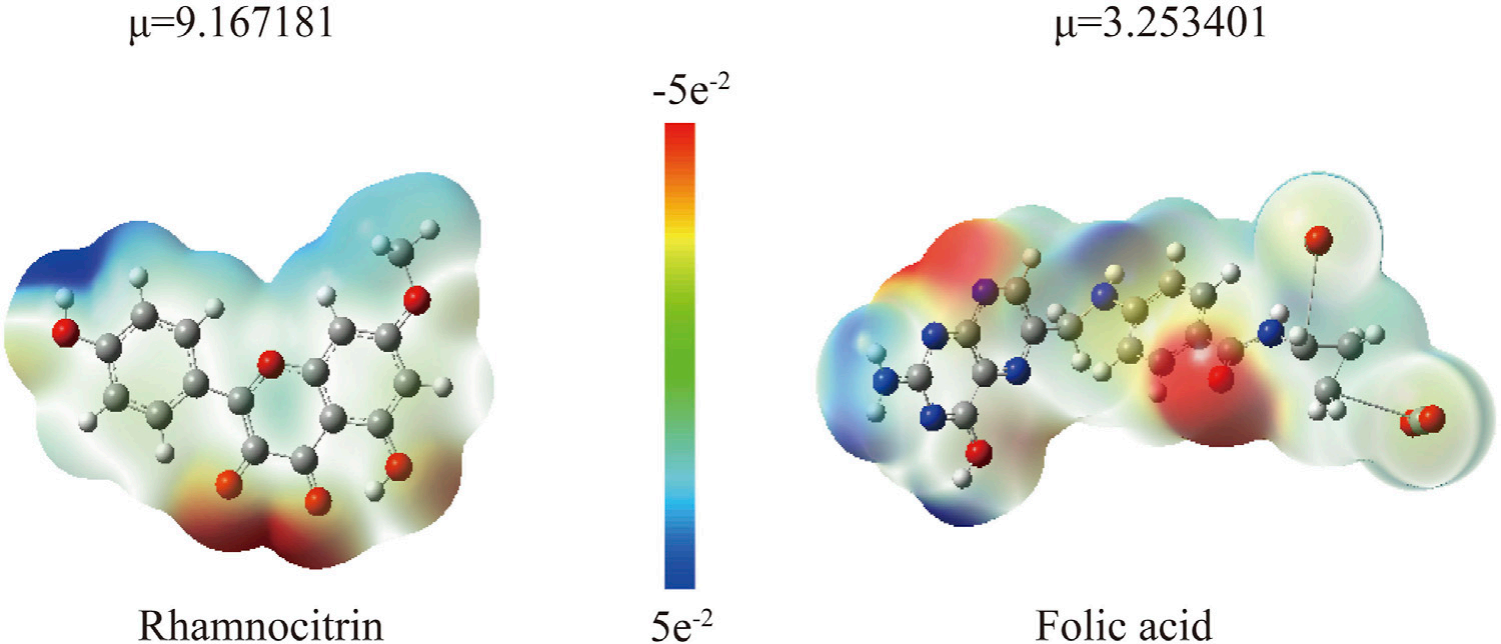
The electrostatic potential map pf Rhamnocitrin and Folic Acid (μ represents the calculated dipole moment) (B).

Through electrostatic potential analysis (Figure 13), it was found that the electron density of rhamnolitrin is primarily concentrated on the carbonyl oxygen, hydroxyl and phenolic hydroxyl groups of the pyrene ring, which facilitates stable binding to the protein binding pocket through hydrogen bonding. The electron density of folic acid is concentrated on the nitrogen atom of the pyrazine ring in the pteridine structure and the carbonyl group of the benzamide. The results of the electrostatic potential analysis are consistent with the aforementioned computational findings, indicating that rhamnolitrin and folic acid achieve stable binding to the GSK3β protein binding pocket through hydrogen bonding and π-π stacking interactions at their active sites.

## 4 Discussion and conclusion

### 4.1 Discussion

This study systematically elucidates the mechanism by which the “Astragalus-Codonopsis” herbal pair regulates GSK3β-mediated insulin signaling through multi-component synergy by the use of multi-level computational biology validation. The network pharmacology revealed that GSK3β is at the core of PPI network, which is highly consistent with the clinical studies linking GSK3β overexpression to insulin resistance. Molecular docking showed that rhamnolitrin and folic acid can bind stably to the binding pocket of GSK3β protein through various interactions such as hydrogen bonds. Alanine virtual scanning further validated the key amino acid residues of protein GSK3β that can bind with these core active ingredients.

The subsequent kinetic simulation revealed the dynamic interaction patterns of rhamnolitrin and folic acid with GSK3β protein in physiological environment. The RMSF and RMSD plots displayed the conformational changes of the complex formed between the compounds and GSK3β protein after the simulation started, with the receptor-ligand complex maintaining stability at the end of the simulation. Furthermore, the hydrogen bonding time diagram and key residue interaction analysis both elucidated the primary chemical interactions between two core components and the GSK3β protein binding pocket. The subsequent torsion distribution histogram visually demonstrated that rhamnolitrin and folic acid were stably inserted into the active cavity of the protein GSK3β, forming a stable “lock-key chelation structure”. Finally, the DFT theoretical calculation analyzed the quantum mechanical properties of rhamnolitrin and folic acid, further clarifying the binding mechanism: the phenolic hydroxyl group and carbonyl oxygen in the structure of rhamnolitrin serve as binding sites, potentially forming hydrogen bonds with the polar amino acid residues of the GSK3β protein. Meanwhile, the delocalized π electrons will also generate π-π accumulation to further stabilize the binding process. The folic acid achieves stable binding with the GSK3β protein through the pteridine part of its structure and the carbonyl oxygen of its amide bond. This may be the key mechanism by which the “Astragalus- Codonopsis” herbal pair reduces (QIAN, X, et al., 2019) GSK3β kinase activity and regulates the insulin receptor signaling pathway, thereby exerting its hypoglycemic effect.

This research has carried out methodological innovation and technical integration based on similar studies in the existing literature. Compared with the conventional research paradigm that combines network pharmacology with molecular docking, this project has constructed a multi-dimensional research system: By integrating technical means such as network pharmacology prediction, molecular docking verification, dynamic analysis of molecular dynamics simulation, key site identification of alanine scanning mutation technology, and DFT theoretical calculation, the multi-target action network and molecular mechanism of drug synergistic therapy for type 2 diabetes mellitus (T2DM) were systematically revealed. This multi-dimensional research strategy not only provides a theoretical basis for clinical transformation but also points out multiple potential targets for the development of new anti-diabetic drugs.

### 4.2 Conclusions

In this study, the molecular mechanism model of “Astragalus-Codonopsis” on the treatment of type 2 diabetes was established: rhamnolitrin and folic acid can bind to GSK3β protein through hydrogen bonding, van der Waals force and hydrophobic interaction, and regulate the insulin receptor signaling pathway to play a pharmacological role in its hypoglycemic effect. The results of this study not only provide a new method for the modernization of TCM compounds, but also lay a theoretical foundation for the development of multi-target hypoglycemic preparations based on natural products.

## Data Availability

The original contributions presented in the study are included in the article/Supplementary Material, further inquiries can be directed to the corresponding authors.
