# Supplementary material for: Investigating the molecular mechanisms of the “Astragalus-Codonopsis” herb pair in treating diabetes: a network pharmacology and bioinformatics approach with molecular docking validation
Source: Front Bioeng Biotechnol. 2025 Jul 7;13:1618575. doi: 10.3389/fbioe.2025.1618575 (PMC12277270; doi:10.3389/fbioe.2025.1618575)
Supplement: Supplementary file 1 [file Table1.docx]

Supplementary Material

**Supplementary Figure 1.** The figure legends are required to have the same font as the main text, 12 point normal Times New Roman, single spaced. Please use a single paragraph for each legend and prepare the figures keeping in mind the PDF layout.

Table S1. Gene names of common target of Astragalus Codonopsis pairs and T2DM

| Gene name | | | | | | |  |
| --- | --- | --- | --- | --- | --- | --- | --- |
| PTGS2 | ADRA2A | PTGS1 | RXRA | NCOA2 | SLC6A2 | GABRA1 | |
| TRPV1 | CHRM1 | CHRM2 | PGR | NOS2 | AR | SCN5A | |
| ESR2 | ESR1 | MAPK14 | GSK3B | NR3C2 | NCOA1 | ADH1C | |
| PYGM | CHRM3 | ADRA1B | F7 | ACHE | RELA | XDH | |
| BCL2 | BAX | CASP9 | CASP3 | ADRB1 | ADRA2C | ADRB2 | |
| ADRA1D | OPRM1 | ADH1A | KCNH2 | CHRM5 | ADRA1A | SLC6A4 | |
| LTA4H | KDR | MET | CTSD | BCHE | IL6 | ADRA2B | |
| CDKN1A | EIF6 | TNF | TP53 | IGF1R | PPARG | ICAM1 | |
| APOB | IL4 | EP300 | ATP5F1B | HSD3B2 | TRPM2 | IL1B | |
| CXCL8 | GSTP1 | INS | LPL | PPARA | HP | PDHX | |
| PPARGC1A | AKT1 | MMP1 | HMOX1 | SLC2A4 | INSR | GOT1 | |
| GOT2 | TPI1 | RXRG | PCNA | RHO | IL10 | PCYT1A | |
| EGFR | BCL2L1 | PLAU | MMP2 | MMP9 | MAPK1 | NFKBIA | |
| ODC1 | TOP1 | HIF1A | ERBB2 | ACACA | PTGER3 | BIRC5 | |
| DUOX2 | IL2 | SERPINE1 | IFNG | NFE2L2 | CD40LG |  | |

Table S2. Molecular docking score table of key active ingredients with target proteins

| Docking score | Core ingredients | PDB ID | Gene name |
| --- | --- | --- | --- |
| 95.4392 | Rhamnocitrin | 1I09 | GSK3B |
| 97.312 | Folic acid | 1I09 | GSK3B |
| The docking failed | isorhamnetin | 1I09 | GSK3B |
| The docking failed | glycitein | 1I09 | GSK3B |
| The docking failed | Calycosin | 1I09 | GSK3B |
| The docking failed | formononetin | 1I09 | GSK3B |
| The docking failed | Mucronulatol | 1I09 | GSK3B |
| The docking failed | tangshenoside III_qt | 1I09 | GSK3B |
| The docking failed | 7-O-methylisomucronulatol | 1I09 | GSK3B |
| The docking failed | tectorigenin | 1I09 | GSK3B |
| The docking failed | (Z)-1-(2,4-dihydroxyphenyl)-3-(4-hydroxyphenyl)prop-2-en-1-one | 1I09 | GSK3B |
| The docking failed | 7-Methoxy-2-methyl isoflavone | 1I09 | GSK3B |

Table S3. The Evaluation of KEGG enrichment analysis results

| Term | Count | P value |
| --- | --- | --- |
| Insulin receptor signaling pathway | 10 | 1.11 |
| Insulin resistance | 12 | 2.98 |
| Th17 cell differentiation | 12 | 2.98 |
| Apoptosis | 13 | 3.09 |
| Measles | 13 | 3.95 |
| Estrogen signaling pathway | 13 | 3.95 |
| Influenza A | 14 | 5.46 |
| Malaria | 9 | 6.63 |
| TNF signaling pathway | 12 | 7.46 |
| T cell receptor signaling pathway | 12 | 9.66 |

Table S4. The Evaluation of GO enrichment analysis results

| Category | Term | Count | % | PValue | Genes |
| --- | --- | --- | --- | --- | --- |
| GOTERM_BP_DIRECT | GO:0043410~positive regulation of MAPK cascade | 16 | 15.38462 | 1E-13 | INSR, ADRA1D, ADRB1, ADRB2, ADRA1B, ADRA2C, ADRA1A, TNF, ADRA2B, ADRA2A, INS, IGF1R, AR, IL6, ERBB2, KDR |
| GOTERM_BP_DIRECT | GO:0010628~positive regulation of gene expression | 21 | 20.19231 | 1.93E-12 | GSK3B, CXCL8, MAPK14, HIF1A, TNF, RELA, SLC6A4, INS, IL4, AR, IL6, IFNG, IL1B, ERBB2, AKT1, PGR, PPARG, APOB, PPARGC1A, TP53, NFE2L2 |
| GOTERM_BP_DIRECT | GO:0071880~adenylate cyclase-activating adrenergic receptor signaling pathway | 8 | 7.692308 | 2.92E-12 | ADRA1D, ADRB1, ADRB2, ADRA1B, ADRA2C, ADRA1A, ADRA2B, ADRA2A |
| GOTERM_BP_DIRECT | GO:0045944~positive regulation of transcription by RNA polymerase II | 29 | 27.88462 | 3.66E-11 | ADRB2, HIF1A, TNF, RELA, EGFR, RXRA, AKT1, EP300, PPARGC1A, RXRG, IL10, NCOA1, NCOA2, MAPK14, ESR1, IL2, ESR2, IL4, NFKBIA, AR, IL6, IFNG, IL1B, PPARG, PGR, PPARA, MET, TP53, NFE2L2 |
| GOTERM_BP_DIRECT | GO:0045429~positive regulation of nitric oxide biosynthetic process | 9 | 8.653846 | 1.05E-10 | IFNG, IL1B, INSR, AKT1, TRPV1, OPRM1, PTGS2, ESR1, TNF |
| GOTERM_BP_DIRECT | GO:0045893~positive regulation of DNA-templated transcription | 22 | 21.15385 | 1.45E-10 | IL10, NCOA1, KCNH2, INSR, HIF1A, ESR1, TNF, EGFR, RELA, ESR2, IL4, AR, IL6, RXRA, IL1B, AKT1, EP300, PPARG, PPARA, PPARGC1A, TP53, NFE2L2 |
| GOTERM_BP_DIRECT | GO:0043066~negative regulation of apoptotic process | 19 | 18.26923 | 1.62E-10 | IL10, GSK3B, CDKN1A, GSTP1, TNF, MMP9, EGFR, IL2, RELA, IGF1R, IL4, IL6, CD40LG, ERBB2, BCL2, BIRC5, AKT1, TP53, BCL2L1 |
| GOTERM_BP_DIRECT | GO:0009410~response to xenobiotic stimulus | 14 | 13.46154 | 1.05E-09 | IL10, CDKN1A, MMP2, PTGS2, SLC6A2, ADRA1A, TNF, RELA, SLC6A4, CASP3, BCL2, PPARG, TOP1, TP53 |
| GOTERM_BP_DIRECT | GO:1902895~positive regulation of miRNA transcription | 9 | 8.653846 | 1.27E-09 | IL10, AR, IL6, PPARG, HIF1A, TNF, TP53, RELA, EGFR |
| GOTERM_BP_DIRECT | GO:0008284~positive regulation of cell population proliferation | 18 | 17.30769 | 1.52E-09 | INSR, ODC1, ADRA1D, EGFR, IL2, RELA, ADRA2A, INS, IGF1R, IL4, AR, IL6, IFNG, IL1B, ERBB2, KDR, BCL2, BIRC5 |
| GOTERM_BP_DIRECT | GO:0051384~response to glucocorticoid | 8 | 7.692308 | 7.97E-09 | IL10, BCHE, IL6, GOT1, CASP3, BCL2, PTGS2, TNF |
| GOTERM_BP_DIRECT | GO:0071222~cellular response to lipopolysaccharide | 12 | 11.53846 | 8.27E-09 | IL10, IL6, CXCL8, NOS2, IL1B, GSTP1, SERPINE1, MAPK14, PTGS2, TNF, MMP9, RELA |
| GOTERM_BP_DIRECT | GO:0008286~insulin receptor signaling pathway | 10 | 9.615385 | 1.11E-08 | GSK3B, INSR, ERBB2, KDR, MAPK1, AKT1, MET, EGFR, IGF1R, INS |
| GOTERM_BP_DIRECT | GO:0048009~insulin-like growth factor receptor signaling pathway | 9 | 8.653846 | 1.11E-08 | AR, INSR, ERBB2, KDR, MAPK1, AKT1, MET, EGFR, IGF1R |
| GOTERM_BP_DIRECT | GO:0010629~negative regulation of gene expression | 14 | 13.46154 | 1.17E-08 | GSK3B, CDKN1A, CXCL8, NOS2, HIF1A, ESR1, TNF, INS, IFNG, KDR, AKT1, PGR, PPARG, XDH |
| GOTERM_BP_DIRECT | GO:0097192~extrinsic apoptotic signaling pathway in absence of ligand | 7 | 6.730769 | 2.29E-08 | IL4, GSK3B, IL1B, BCL2, BAX, IL2, BCL2L1 |
| GOTERM_BP_DIRECT | GO:0051091~positive regulation of DNA-binding transcription factor activity | 9 | 8.653846 | 2.36E-08 | IL10, IL6, IL1B, EP300, AKT1, ESR1, TNF, PPARGC1A, ESR2 |
| GOTERM_BP_DIRECT | GO:0001666~response to hypoxia | 11 | 10.57692 | 3.68E-08 | CASP9, F7, NOS2, PLAU, CASP3, MMP2, EP300, PPARA, HIF1A, TNF, SLC6A4 |
| GOTERM_BP_DIRECT | GO:0007197~adenylate cyclase-inhibiting G protein-coupled acetylcholine receptor signaling pathway | 5 | 4.807692 | 5.14E-08 | CHRM2, CHRM3, CHRM1, CHRM5, OPRM1 |
| GOTERM_BP_DIRECT | GO:0007623~circadian rhythm | 8 | 7.692308 | 8.77E-08 | GSK3B, F7, NOS2, EP300, TOP1, TNF, EGFR, SLC6A4 |
| GOTERM_BP_DIRECT | GO:0071356~cellular response to tumor necrosis factor | 9 | 8.653846 | 1.34E-07 | NFKBIA, CXCL8, MAPK1, AKT1, TRPV1, SLC2A4, MAPK14, RELA, NFE2L2 |
| GOTERM_BP_DIRECT | GO:0035357~peroxisome proliferator activated receptor signaling pathway | 5 | 4.807692 | 1.53E-07 | NCOA1, NCOA2, RXRA, PPARG, PPARA |
| GOTERM_BP_DIRECT | GO:0043065~positive regulation of apoptotic process | 13 | 12.5 | 1.65E-07 | NCOA1, TRPV1, PTGS2, TNF, MMP9, CASP9, IL6, BCL2, BAX, PPARG, CTSD, TP53, BCL2L1 |
| GOTERM_BP_DIRECT | GO:0048013~ephrin receptor signaling pathway | 8 | 7.692308 | 3.38E-07 | MMP2, INSR, ERBB2, KDR, MET, MMP9, EGFR, IGF1R |
| GOTERM_BP_DIRECT | GO:0030335~positive regulation of cell migration | 12 | 11.53846 | 3.39E-07 | IL4, F7, PLAU, IL1B, MMP2, INSR, KDR, AKT1, EGFR, ADRA2A, IGF1R, INS |
| GOTERM_BP_DIRECT | GO:0032355~response to estradiol | 8 | 7.692308 | 3.97E-07 | NCOA1, CASP9, F7, PCNA, CASP3, PTGS2, ESR1, SLC6A4 |
| GOTERM_BP_DIRECT | GO:0048661~positive regulation of smooth muscle cell proliferation | 7 | 6.730769 | 4.04E-07 | IL6, HMOX1, AKT1, PTGS2, TNF, EGFR, IGF1R |
| GOTERM_BP_DIRECT | GO:0007186~G protein-coupled receptor signaling pathway | 20 | 19.23077 | 4.2E-07 | CHRM2, CHRM3, CHRM1, CXCL8, INSR, PTGER3, ADRA1D, ADRA1B, ADRA2C, ESR1, ADRA1A, ADRA2B, IL2, ADRA2A, INS, AR, RHO, AKT1, PGR, PPARG |
| GOTERM_BP_DIRECT | GO:0007267~cell-cell signaling | 11 | 10.57692 | 4.37E-07 | AR, IL1B, ADRA1D, PGR, ADRA1B, ADRA2C, ADRA1A, ADRA2B, ESR2, IL2, INS |
| GOTERM_BP_DIRECT | GO:1902894~negative regulation of miRNA transcription | 6 | 5.769231 | 4.89E-07 | PPARG, PPARA, HIF1A, ESR1, TNF, RELA |
| GOTERM_BP_DIRECT | GO:0010507~negative regulation of autophagy | 7 | 6.730769 | 7.64E-07 | IL10, BCL2, EP300, AKT1, ADRA1A, MET, BCL2L1 |
| GOTERM_BP_DIRECT | GO:0038084~vascular endothelial growth factor signaling pathway | 7 | 6.730769 | 9.3E-07 | INSR, ERBB2, KDR, MET, RELA, EGFR, IGF1R |
| GOTERM_BP_DIRECT | GO:0031622~positive regulation of fever generation | 4 | 3.846154 | 1.44E-06 | IL1B, PTGER3, PTGS2, TNF |
| GOTERM_BP_DIRECT | GO:2000352~negative regulation of endothelial cell apoptotic process | 6 | 5.769231 | 1.45E-06 | IL10, IL4, SERPINE1, KDR, ICAM1, NFE2L2 |
| GOTERM_BP_DIRECT | GO:0071392~cellular response to estradiol stimulus | 6 | 5.769231 | 1.66E-06 | IL10, MMP2, ESR1, ESR2, EGFR, IGF1R |
| GOTERM_BP_DIRECT | GO:0071466~cellular response to xenobiotic stimulus | 7 | 6.730769 | 1.92E-06 | KCNH2, PCNA, NOS2, IL1B, TP53, EGFR, NFE2L2 |

Table S5. Topological analysis of the PPI network

| Degree | degree.layout | name | selected | shared name |
| --- | --- | --- | --- | --- |
| 104 | 104 | GSK3B | FALSE | GSK3B |
| 64 | 64 | IL1B | FALSE | IL1B |
| 63 | 63 | PTGS2 | FALSE | PTGS2 |
| 61 | 61 | ESR1 | FALSE | ESR1 |
| 60 | 60 | TP53 | FALSE | TP53 |
| 59 | 59 | EGFR | FALSE | EGFR |
| 57 | 57 | IL6 | FALSE | IL6 |
| 57 | 57 | CASP3 | FALSE | CASP3 |
| 57 | 57 | PPARG | FALSE | PPARG |
| 56 | 56 | TNF | FALSE | TNF |
| 55 | 55 | BCL2 | FALSE | BCL2 |
| 53 | 53 | HIF1A | FALSE | HIF1A |
| 52 | 52 | INS | FALSE | INS |
| 51 | 51 | MMP9 | FALSE | MMP9 |
| 50 | 50 | IL10 | FALSE | IL10 |
| 48 | 48 | CXCL8 | FALSE | CXCL8 |
| 47 | 47 | ERBB2 | FALSE | ERBB2 |
| 47 | 47 | IFNG | FALSE | IFNG |
| 45 | 45 | RELA | FALSE | RELA |
| 44 | 44 | ICAM1 | FALSE | ICAM1 |
| 42 | 42 | BCL2L1 | FALSE | BCL2L1 |
| 42 | 42 | MMP2 | FALSE | MMP2 |
| 42 | 42 | HMOX1 | FALSE | HMOX1 |
| 42 | 42 | PPARA | FALSE | PPARA |
| 41 | 41 | MAPK14 | FALSE | MAPK14 |
| 41 | 41 | NFKBIA | FALSE | NFKBIA |
| 41 | 41 | AKT1 | FALSE | AKT1 |
| 41 | 41 | NFE2L2 | FALSE | NFE2L2 |
| 41 | 41 | PPARGC1A | FALSE | PPARGC1A |
| 40 | 40 | SERPINE1 | FALSE | SERPINE1 |
| 39 | 39 | EP300 | FALSE | EP300 |
| 39 | 39 | MAPK1 | FALSE | MAPK1 |
| 38 | 38 | CDKN1A | FALSE | CDKN1A |
| 38 | 38 | CASP9 | FALSE | CASP9 |
| 37 | 37 | IL2 | FALSE | IL2 |
| 37 | 37 | IL4 | FALSE | IL4 |
| 36 | 36 | KDR | FALSE | KDR |
| 35 | 35 | IGF1R | FALSE | IGF1R |
| 35 | 35 | ESR2 | FALSE | ESR2 |
| 35 | 35 | PGR | FALSE | PGR |
| 33 | 33 | AR | FALSE | AR |
| 33 | 33 | SLC2A4 | FALSE | SLC2A4 |
| 31 | 31 | PLAU | FALSE | PLAU |
| 30 | 30 | NOS2 | FALSE | NOS2 |
| 28 | 28 | APOB | FALSE | APOB |
| 27 | 27 | ADRB2 | FALSE | ADRB2 |
| 26 | 26 | MMP1 | FALSE | MMP1 |
| 26 | 26 | XDH | FALSE | XDH |
| 25 | 25 | PTGS1 | FALSE | PTGS1 |
| 24 | 24 | MET | FALSE | MET |
| 23 | 23 | CTSD | FALSE | CTSD |
| 23 | 23 | GOT2 | FALSE | GOT2 |
| 21 | 21 | NCOA2 | FALSE | NCOA2 |
| 21 | 21 | NCOA1 | FALSE | NCOA1 |
| 21 | 21 | RXRA | FALSE | RXRA |
| 21 | 21 | CD40LG | FALSE | CD40LG |
| 20 | 20 | GSTP1 | FALSE | GSTP1 |
| 18 | 18 | OPRM1 | FALSE | OPRM1 |
| 18 | 18 | ACHE | FALSE | ACHE |
| 18 | 18 | LPL | FALSE | LPL |
| 17 | 17 | HP | FALSE | HP |
| 17 | 17 | BAX | FALSE | BAX |
| 15 | 15 | NR3C2 | FALSE | NR3C2 |
| 15 | 15 | BIRC5 | FALSE | BIRC5 |
| 15 | 15 | SLC6A4 | FALSE | SLC6A4 |
| 14 | 14 | TRPV1 | FALSE | TRPV1 |
| 14 | 14 | TPI1 | FALSE | TPI1 |
| 13 | 13 | BCHE | FALSE | BCHE |
| 13 | 13 | SLC6A2 | FALSE | SLC6A2 |
| 13 | 13 | CHRM2 | FALSE | CHRM2 |
| 12 | 12 | INSR | FALSE | INSR |
| 11 | 11 | DUOX2 | FALSE | DUOX2 |
| 11 | 11 | ACACA | FALSE | ACACA |
| 10 | 10 | ADRA2A | FALSE | ADRA2A |
| 10 | 10 | CHRM1 | FALSE | CHRM1 |
| 9 | 9 | PYGM | FALSE | PYGM |
| 9 | 9 | ADRA2B | FALSE | ADRA2B |
| 8 | 8 | RXRG | FALSE | RXRG |
| 8 | 8 | F7 | FALSE | F7 |
| 8 | 8 | ODC1 | FALSE | ODC1 |
| 8 | 8 | ADRB1 | FALSE | ADRB1 |
| 7 | 7 | PTGER3 | FALSE | PTGER3 |
| 7 | 7 | TOP1 | FALSE | TOP1 |
| 7 | 7 | RHO | FALSE | RHO |
| 7 | 7 | ADRA1D | FALSE | ADRA1D |
| 7 | 7 | ADRA1B | FALSE | ADRA1B |
| 7 | 7 | ADRA1A | FALSE | ADRA1A |
| 6 | 6 | PCNA | FALSE | PCNA |
| 6 | 6 | PDHX | FALSE | PDHX |
| 6 | 6 | ATP5F1B | FALSE | ATP5F1B |
| 6 | 6 | CHRM3 | FALSE | CHRM3 |
| 5 | 5 | GABRA1 | FALSE | GABRA1 |
| 4 | 4 | LTA4H | FALSE | LTA4H |
| 4 | 4 | GOT1 | FALSE | GOT1 |
| 4 | 4 | ADH1A | FALSE | ADH1A |
| 3 | 3 | CHRM5 | FALSE | CHRM5 |
| 3 | 3 | HSD3B2 | FALSE | HSD3B2 |
| 3 | 3 | KCNH2 | FALSE | KCNH2 |
| 3 | 3 | ADH1C | FALSE | ADH1C |
| 2 | 2 | SCN5A | FALSE | SCN5A |
| 2 | 2 | EIF6 | FALSE | EIF6 |
| 2 | 2 | GSK3BR | FALSE | GSK3BR |
| 1 | 1 | TRPM2 | FALSE | TRPM2 |
| 1 | 1 | PCYT1A | FALSE | PCYT1A |
